# Performance of deep learning-based segmentation of soft tissue sarcoma by MRI sequence, tumor type and location

**DOI:** 10.1007/s00256-026-05178-3

**Published:** 2026-03-04

**Authors:** Linkai Peng, Laetitia Perronne, Nicolò Gennaro, Ahmad Pour Rashidi, Zuzanna Kobus, Mirinae Seo, Amir A. Borhani, Linda Kelahan, Kamal Subedi, Hatice Savas, Ryan Avery, Tugce Agirlar Trabzonlu, Chase Krumpelman, Spyridon Bakas, Akhil Chawla, Sean Sachdev, Pedro Hermida de Viveiros, Seth M. Pollack, Ulas Bagci, Yuri S. Velichko

**Affiliations:** 1 Department of Radiology, Northwestern University Feinberg School of Medicine, Chicago, IL USA; 2 Department of Pathology, Indiana University School of Medicine, Indianapolis, IN USA; 3 Department of Surgery, Northwestern University Feinberg School of Medicine, Chicago, IL USA; 4 Robert H. Lurie Comprehensive Cancer Center, Northwestern University Feinberg School of Medicine, Chicago, IL USA; 5 Department of Radiation Oncology, Northwestern University Feinberg School of Medicine, Chicago, IL USA; 6 Department of Medicine, Northwestern University Feinberg School of Medicine, Chicago, IL USA

**Keywords:** Soft tissue sarcoma, MRI, Segmentation, Deep learning, Histology, Anatomic location

## Abstract

**Objective:**

Soft tissue sarcomas (STS) are a rare and heterogeneous group of tumors that pose a significant challenge for surgical planning. This study evaluated the performance of a deep learning model for automated STS segmentation on preoperative MRI, focusing on how different MRI sequences, anatomical locations, and histological subtypes influence model accuracy.

**Materials and methods:**

We retrospectively analyzed 299 patients with biopsy-proven leiomyosarcoma (*n* = 67), myxofibrosarcoma (*n* = 55), myxoid liposarcoma (*n* = 60), undifferentiated pleomorphic sarcoma (*n* = 70), and intramuscular myxoma (*n* = 47) with pre-treatment MRI (2004–2022). Tumors were manually segmented on fat-suppressed, contrast-enhanced T1-weighted, and fat-suppressed T2-weighted sequences. Separate 3D nnU-Net models were trained for each MRI sequence and combination. Performance was evaluated using Dice, F2 score, average symmetric surface distance (ASSD), and 95th percentile Hausdorff distance (HD95).

**Results:**

Overall, single-sequence T1 models outperformed multi-modal and multi-plane approaches. The T1 axial model achieved the best volumetric accuracy (median F2 score 0.91, Dice 0.89), while the T1 sagittal model provided superior boundary delineation (ASSD 2.1 mm, HD95 4.3 mm). Performance varied by anatomical site: T1 sequences were optimal for tumors in the extremities, whereas T2 sequences were more effective for abdominal and pelvic lesions. Accuracy also varied by histology, with the highest performance for myxofibrosarcoma and myxoid liposarcoma (F2 up to 0.94) and the lowest for leiomyosarcoma (F2 up to 0.88). Unexpectedly, multi-modal fusion of T1 and T2 images did not improve results and often degraded boundary accuracy.

**Conclusion:**

Deep learning models achieve high accuracy for STS segmentation. However, their performance is critically dependent on tumor location and histology.

**Supplementary information:**

The online version contains supplementary material available at 10.1007/s00256-026-05178-3.

## Introduction

Soft tissue sarcomas (STS) are a rare and heterogeneous group of malignant mesenchymal tumors [1]. While they account for less than 1% of all adult cancers, they represent more than 13,000 new diagnoses annually in the USA [2]. With over 70 distinct histologic subtypes arising in virtually any anatomical location, this intrinsic heterogeneity results in highly variable imaging appearances and clinical behaviors, presenting significant challenges for clinicians in both diagnosis and treatment planning [3–9].

Accurate delineation of tumor boundaries is critical for STS management. For surgeons, it ensures complete resection with negative margins—the foundation of curative-intent therapy. For radiation oncologists, it defines the gross tumor volume (GTV), directly influencing dose delivery and sparing of healthy tissue. However, infiltrative growth and proximity to vital structures often obscure tumor margins, complicating efforts to balance oncologic control with functional preservation [10–12]. Reliable and reproducible tumor delineation is also fundamental for assessing tumor biology and treatment response [13].

Magnetic resonance imaging (MRI) is the modality of choice for the local staging and pre-treatment evaluation of most STS due to its superior soft tissue contrast [14]. In clinical practice, radiologists typically rely on a combination of fat-suppressed T2-weighted and contrast-enhanced T1-weighted sequences to delineate tumor extent. However, the process of manual tumor segmentation on MRI is time-consuming and inherently subject to inter-observer variability. Given the irregular morphology, infiltrative growth pattern, and heterogeneous enhancement characteristic of different sarcoma subtypes, this variability has been reported to reach up to 20% of the tumor volume, resulting in a difference of as much as 1–2 cm at the margins [15]. Such variability may directly impact surgical and radiotherapy planning, potentially resulting in incomplete resections, local recurrence, or the unnecessary sacrifice of healthy tissue. In extreme cases, this uncertainty at the tumor margin can determine the choice between limb-salvage and amputation [16]. In this context, deep learning-based segmentation represents a promising approach to improve efficiency, reproducibility, and standardization in tumor delineation.

Although deep learning-based segmentation models have achieved substantial success in automating image delineation for many common cancers, their application to STS remains limited due to the rarity, histologic diversity, and complex anatomical presentation of these tumors [17–23]. Previous studies have shown the potential of deep learning for soft tissue sarcoma segmentation, but remain limited in scope. Peeken et al. developed a robust MRI-based model for extremity STS, yet did not assess the influence of MRI sequences, imaging planes, or histological variability [22]. Marin et al. proposed an innovative approach for sarcoma gross tumor volume delineation on CT images, primarily for radiotherapy planning rather than MRI-based segmentation [15]. Zheng et al. introduced a multi-modal UNet model for thigh sarcomas, but their analysis relied on a small single-site cohort [24]. Developing a robust, automated tool for STS could therefore provide significant clinical value in preoperative planning, intraoperative navigation, and multidisciplinary decision-making. However, for such a tool to achieve clinical reliability and generalizability, it must effectively address the profound biological and imaging heterogeneity inherent to STS. This underscores a critical unresolved question: which MRI-derived data and modeling strategies most accurately capture the full spectrum of STS phenotypes, and variability across histological subtypes and anatomical sites influences model performance and clinical applicability.

This study aims to establish a clinically robust strategy for automated STS segmentation by examining how model performance is influenced by two key determinants: the technical selection of MRI input and the biological heterogeneity of the disease. Using the nnU-Net framework, we systematically evaluated the impact of (1) MRI sequence (fat-suppressed contrast-enhanced T1-weighted vs. T2-weighted), (2) imaging plane (axial, coronal, and sagittal), and (3) data fusion strategies on segmentation accuracy. To evaluate clinical generalizability, we further analyzed model performance across distinct histological subtypes and anatomical locations, providing a comprehensive characterization of the framework’s strengths and limitations in a real-world context. Ultimately, this work seeks to define the parameters that most critically determine segmentation performance in STS, thereby informing the development of standardized, reproducible, and clinically deployable AI-based workflows for sarcoma imaging.

## Materials and methods

### Study population

This retrospective study received Institutional Review Board (IRB) approval with waivers for patient informed consent and Health Insurance Portability and Accountability Act (HIPAA) authorization.

**Fig. 1:**
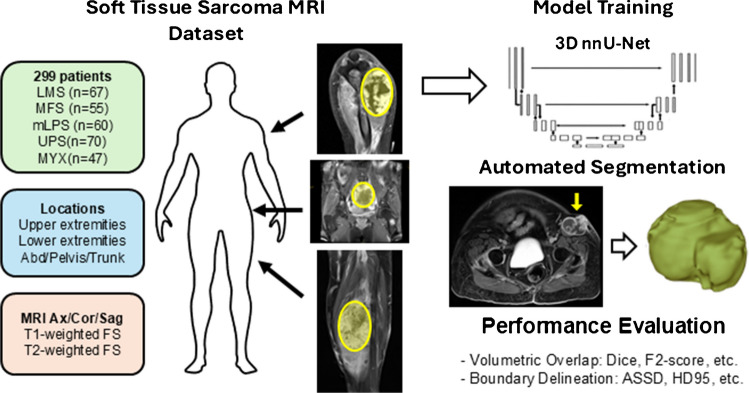
Schematic of the soft tissue tumor automated segmentation workflow

A patient cohort was assembled by searching the institutional electronic data warehouse (EDW) for patients with biopsy-proven soft tissue sarcoma (STS) who underwent pre-treatment magnetic resonance imaging (MRI) of the primary tumor between January 2004 and December 2022. Following the search, anonymized MRI scans were retrieved from the institutional picture archiving and communication system (PACS). Patients were included if their scan had at least one T1-weighted fat-saturated contrast-enhanced (T1 FS CE) or fat-saturated T2-weighted (T2 FS) sequence. Per standard clinical protocols, these sequences were routinely acquired in the axial, coronal, and sagittal planes with sub-millimeter in-plane resolution. This multi-planar approach was crucial for accurately delineating the tumor’s three-dimensional anatomy, heterogeneity, and relationship with adjacent structures, especially given the frequently elongated morphology of sarcomas. The study workflow is summarized in Fig. 1.

From an initial group of approximately 780 patients, a final cohort of 299 was selected. To ensure reliable model training and performance evaluation for each histology, we included only the five largest mesenchymal tumor subgroups: leiomyosarcoma (LMS; *n* = 67), myxofibrosarcoma (MFS; *n* = 55), myxoid liposarcoma (mLPS; *n* = 60), undifferentiated pleomorphic sarcoma (UPS; *n* = 70), and intramuscular myxoma (MYX; *n* = 47). The inclusion of MYX, a benign sarcoma mimic, provided an important control cohort for evaluating model specificity. These subtypes represent the most prevalent and clinically relevant entities encountered in our practice and offered well-balanced case distribution for model development.

### MRI acquisition, image preprocessing, and tumor segmentation

The final MRI dataset comprised 248 T1-weighted axial, 208 T1-weighted coronal, 180 T1-weighted sagittal, 224 T2-weighted axial, 217 T2-weighted coronal, and 171 T2-weighted sagittal scans. To ensure uniformity and reproducibility across the dataset, all scans underwent standardized preprocessing procedures [25]. First, N4 bias field correction was applied to mitigate intensity non-uniformities arising from magnetic field inhomogeneities and scanner imperfections [26]. Next, image intensities were normalized using Z-score standardization, harmonizing signal distribution across scans and patients [27]. Finally, all images were resampled to an isotropic voxel size of 1 mm^3^ using fifth-degree Lagrangian polynomial interpolation [28] to standardize spatial resolution and facilitate 3D convolutional processing.

Manual tumor segmentation was performed using LIFEx (version 7.6, Orsay, France) [28] by trained radiologists (L.P., N.G., M.S.) experienced in sarcoma imaging and a fourth-year medical student (Z.K.) trained in sarcoma image analysis. Lesions were segmented volumetrically across all available fat-saturated, contrast-enhanced T1-weighted and fat-saturated T2-weighted MRI sequences across all planes. All segmentations underwent independent cross-review by two senior radiologists (L.P. and N.G.) to ensure accuracy, anatomical completeness, and inter-rater consistency.

### Tumor segmentation model

We utilized a 3D full-resolution nnU-Net, a self-configuring deep learning framework based on the U-Net architecture that automatically optimizes its own network depth, patch size, and batch size for a given dataset [29]. For this study, the dataset was partitioned into training (70%) and testing (30%) sets, with model development performed on the training set using fivefold cross-validation. To determine the optimal input strategy, we trained separate models for each imaging plane (axial, coronal, and sagittal) across three distinct sequence configurations: T1-weighted only, T2-weighted only, and combined T1-weighted and T2-weighted (T1 + T2) inputs. This design enabled assessment of both technical factors (input modality and orientation) and information fusion strategies on segmentation performance.

During training, we employed extensive data augmentation, including random rotations, scaling, Gaussian noise, gamma correction, and mirroring [30]. The nnU-Net framework automatically configured a patch size of 160 × 112 × 128 voxels and a batch size of 2 to optimize GPU memory usage. Models were trained for 1000 epochs using a combined Dice and cross-entropy loss function. The network optimizer was stochastic gradient descent (SGD) with Nesterov momentum (0.99) [31], an initial learning rate of 0.01, and a weight decay of 3 × 10⁻^5^. At inference, predictions were generated using a sliding window approach with Gaussian importance weighting to ensure smooth segmentation maps.

### Statistical analysis

Model performance was comprehensively evaluated using both volumetric overlap and boundary delineation accuracy metrics. For volumetric overlap, we computed the Dice Similarity Coefficient (Dice), Precision, Recall, and the F2 score. For boundary accuracy, we used the Average Symmetric Surface Distance (ASSD) and the 95th Percentile Hausdorff Distance (HD95). All performance metrics were summarized using the median and interquartile range (IQR) given the non-normal distribution of the data. For model selection, the F2 score was chosen as the primary metric and ASSD as a secondary one.

We selected the F2 score as our primary metric because, from a clinical standpoint, under-segmenting a tumor (a false negative at the voxel level) is a more severe error than slight over-segmentation. Inadvertently leaving residual tumor behind due to under-segmentation can lead directly to local recurrence, whereas minor overestimation might result in a slightly larger but still safe surgical margin. This prioritization aligns with the critical surgical objective of achieving a complete tumor resection with negative margins. The model with the highest median F2 score and the smallest median ASSD was selected as the optimal performer for each category (tumor subtype, location, and overall).

## Results

### Patient and tumor characteristics

The demographic and clinical characteristics of the 299-patient cohort are summarized in Table 1. The cohort included five primary soft tissue sarcoma histotypes: leiomyosarcoma (LMS), myxofibrosarcoma (MFS), myxoid liposarcoma (mLPS), undifferentiated pleomorphic sarcoma (UPS), and myxoma (MYX). The patient population was predominantly female (57%), with an average age that varied by subtype (range: 47 to 66 years). Tumors were mostly located in the lower extremities (72%), and the most frequent tumor grade was grade 3 (42%). At the time of the evaluation, 30% of patients presented with metastatic disease.

**Table 1:** Patient characteristics

Histotype	LMS	MFS	mLPS	UPS	MYX	Total
Subject count (299 total)						
Female	40 (60%)	25 (45%)	28 (47%)	42 (60%)	34 (72%)	169 (57%)
Male	27 (40%)	30 (55%)	32 (53%)	28 (40%)	13 (28%)	130 (43%)
Total	67	55	60	70	47	299
Age	59 ± 15	66 ± 12.5	47 ± 21.5	62 ± 16	54 ± 17	58 ± 16
Grade						
G1	6 (9%)	7 (13%)	23 (38%)	0 (0%)	0 (0%)	36 (12%)
G2	17 (25%)	20 (36%)	11 (18%)	13 (19%)	0 (0%)	61 (20%)
G3	34 (51%)	25 (45%)	10 (17%)	56 (80%)	0 (0%)	125 (42%)
N/A	10 (15%)	3 (5%)	6 (10%)	1 (1%)	47 (100%)	67 (22%)
Tumor location						
Upper extremity	9 (13%)	12 (22%)	1 (2%)	8 (11%)	9 (19%)	39 (13%)
Lower extremity	34 (51%)	40 (73%)	53 (88%)	54 (77%)	33 (70%)	214 (72%)
Abdomen/pelvis	21 (31%)	1 (2%)	5 (8%)	5 (7%)	5 (11%)	37 (12%)
Trunk	1 (1%)	2 (4%)	1 (2%)	3 (4%)	0 (0%)	7 (2%)
Other	2 (3%)	0 (0%)	0 (0%)	0 (0%)	0 (0%)	2 (1%)
Metastasis						
Yes	38 (57%)	11 (20%)	13 (22%)	29 (41%)	0 (0%)	91 (30%)
No	29 (43%)	44 (80%)	47 (78%)	41 (59%)	0 (0%)	161 (54%)
N/A	0 (0%)	0 (0%)	0 (0%)	0 (0%)	47 (100%)	47 (16%)

### Comparative performance of MRI input strategies

**Fig. 2:**
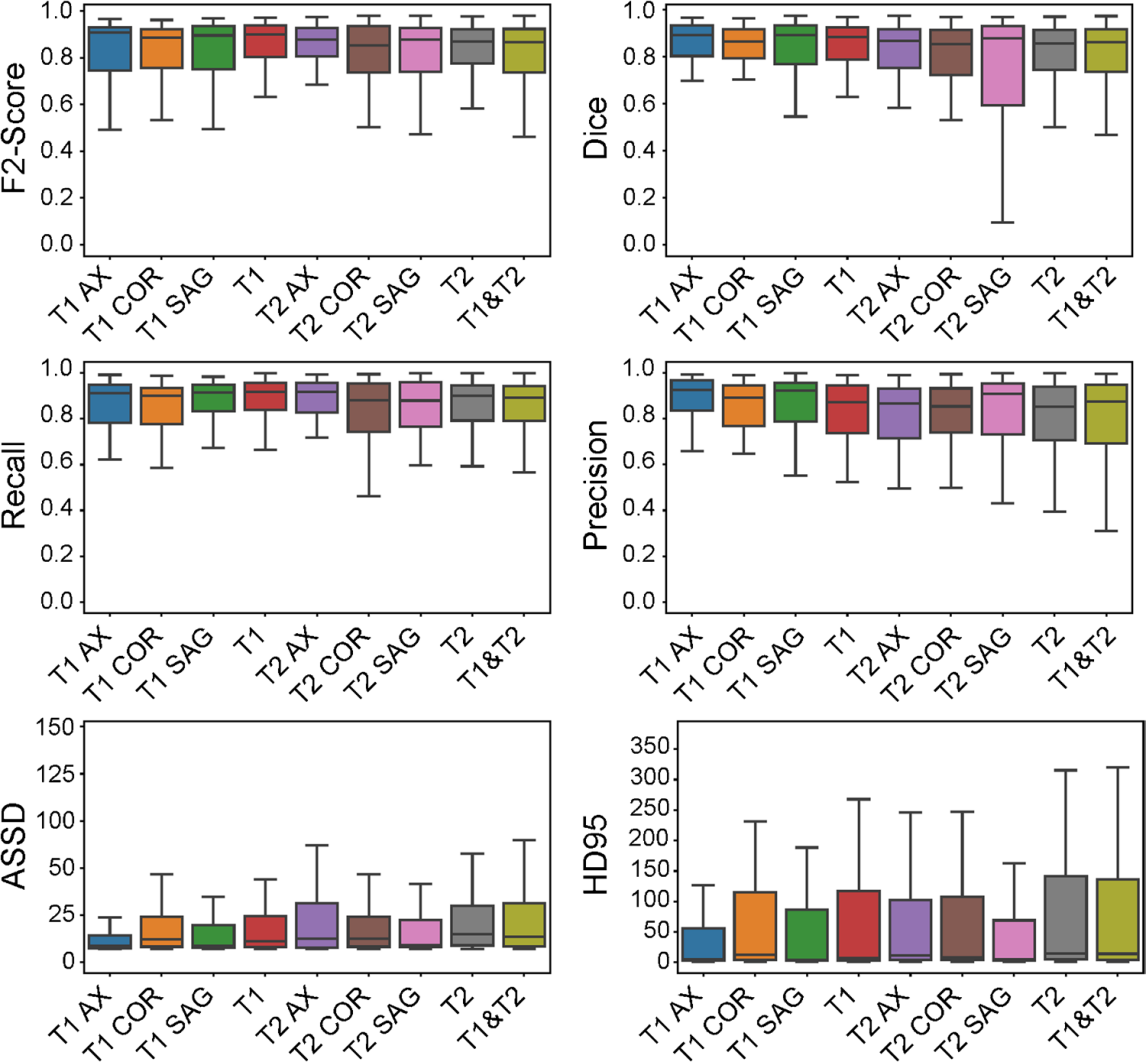
Box plots summarizing the performance of the nine different MRI input strategies across the entire test cohort. Each box represents the interquartile range, the central line indicates the median, and the whiskers show the data range

**Fig. 3:**
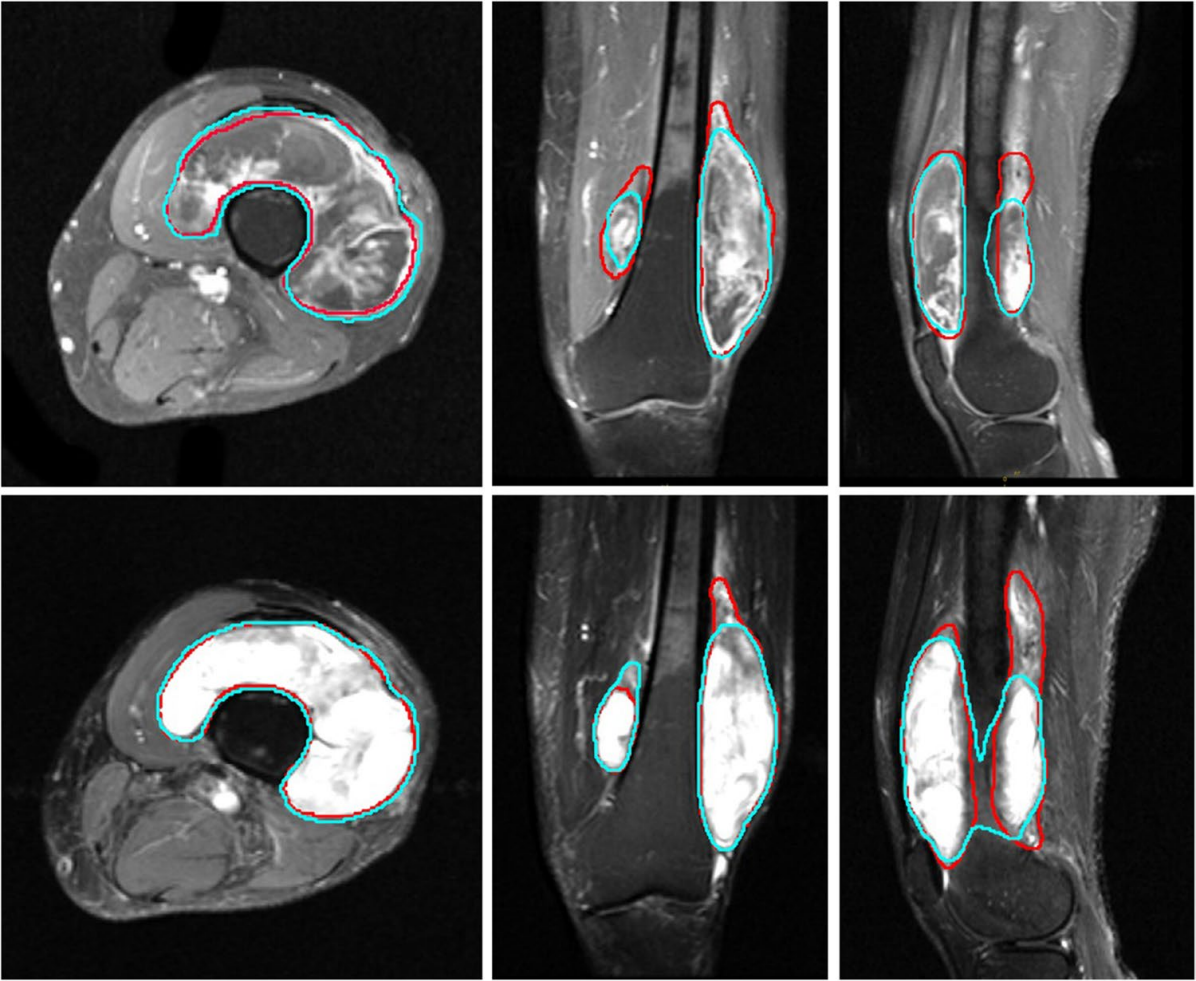
Representative T1- and T2-weighted MR images of a myxoid liposarcoma in the lower extremity are shown in axial (left), coronal (middle), and sagittal (right) planes. The ground truth segmentation, manually delineated by a radiologist, is outlined in red, while the automated prediction from the respective single-plane model is outlined in cyan

The study evaluated nine distinct nnU-Net model configurations for STS segmentation across five histological subtypes and three anatomical locations. Aggregated performance metrics—averaged across all sarcoma subtypes and tumor locations—revealed a clear hierarchy among the different MRI input strategies (Fig. 2). The T1-weighted axial (T1 AX) model emerged as a top performer, achieving the highest median F2 score (0.91) and median Dice score (0.89), demonstrating strong overall segmentation accuracy. The T1-weighted sagittal (T1 SAG) model performed comparably, with a median F2 score of 0.90 and a median Dice score of 0.89. Regarding boundary accuracy, the T1 AX model had a median HD95 of 5.6 mm and a median ASSD of 2.4 mm. While these are strong results, the T1 SAG model outperformed it with a median HD95 of 4.3 mm and a median ASSD of 2.1 mm, securing the top spot for boundary accuracy (Fig. 3).

Among the T2-weighted models, the T2 SAG and T2 AX configurations demonstrated comparable volumetric accuracy (median F2 score of 0.88, while their median Dice scores were 0.88 and 0.87, respectively). However, the difference in boundary accuracy was more pronounced; the T2 SAG model exhibited superior boundary delineation (ASSD = 2.7 mm; HD95 of 5.1 mm) compared to the T2 AX model (ASSD = 8.4 mm; HD95 of 14.9 mm).

**Fig. 4:**
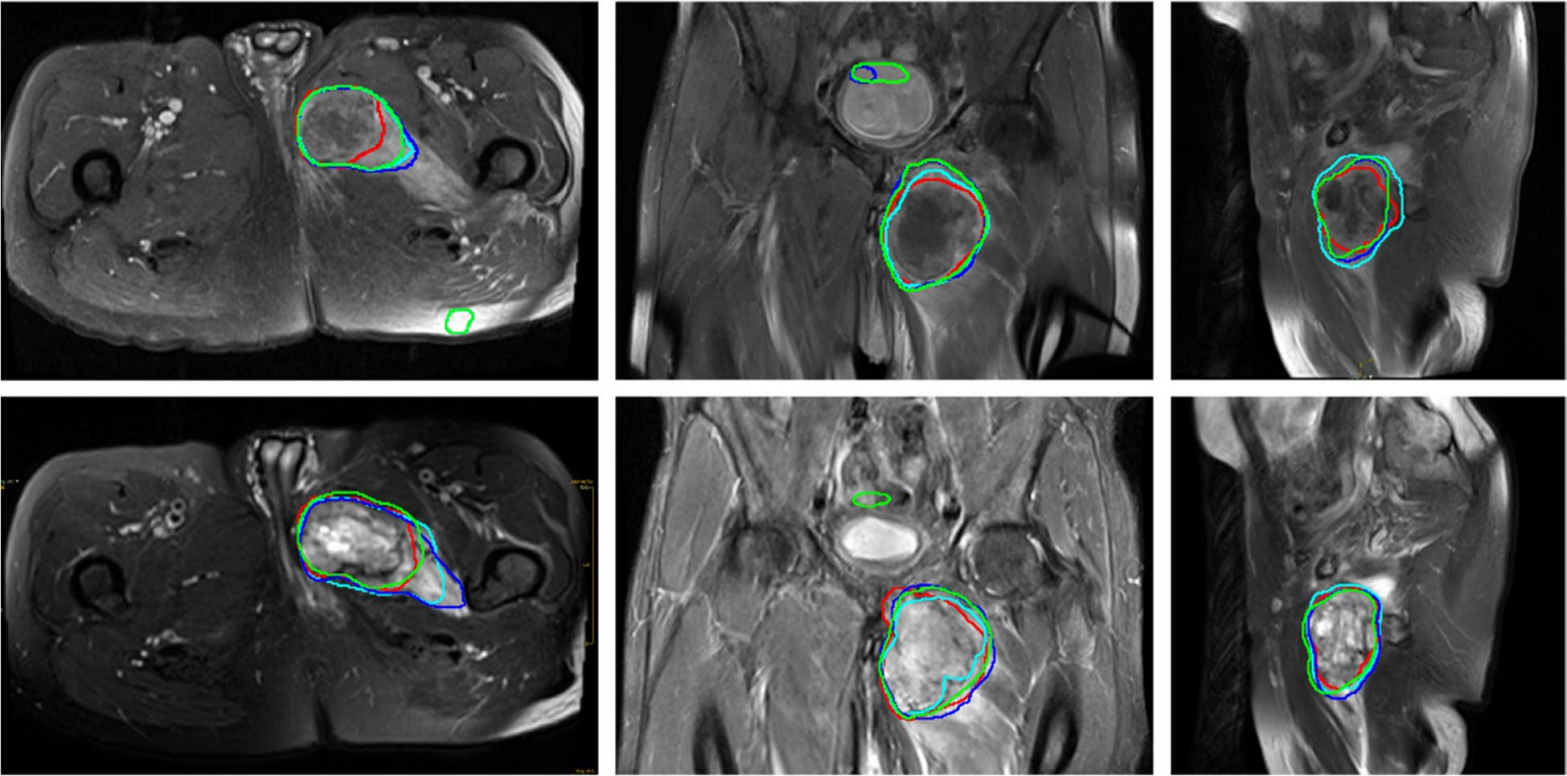
Representative T1- and T2-weighted MR images of an undifferentiated pleomorphic sarcoma in the lower extremity are shown in axial (left), coronal (middle), and sagittal (right) planes. The ground truth segmentation, manually delineated by a radiologist, is outlined in red, while automated predictions are shown from the respective single-plane model (cyan), the multi-plane T1 or T2 model (blue), and the multi-modal T1 and T2 model (green)

While the multi-planar T1 model (T1 AX/COR/SAG) showed strong performance with a median F2 score of 0.90, a median Dice score of 0.88, a median ASSD of 2.4 mm, and a median HD95 of 5.56 mm, it did not outperform the best single-plane T1 models (Fig. 4). Similarly, the multi-planar T2 model (T2 AX/COR/SAG) achieved a median F2 score of 0.87 and a median Dice score of 0.86 but demonstrated subpar boundary accuracy with a median ASSD of 8.4 mm and a median HD95 of 14.9 mm. Finally, the combined multi-modal model (T1 and T2), with a median F2 score of 0.88, a median Dice score of 0.88, a median ASSD of 2.7 mm, and a median HD95 of 5.1 mm, was also suboptimal compared to the best single-plane models. A summary of the aggregated performance for all model configurations is presented in Table S1 (Supplementary materials).

### Performance analysis by anatomical location

A detailed breakdown of the model’s performance by anatomical location reveals sequence- and plane-specific strengths (Table 2). The T1 SAG model was the top performer for tumors in the upper extremities, which made up the majority of the study’s cohort. In this region, it achieved a high median F2 score of 0.85 and a median Dice score of 0.88, along with excellent boundary accuracy with a median ASSD of 1.5 mm and a median HD95 of 4.24 mm. For the lower extremities, the T1 AX model proved to be the most robust performer, yielding a median F2 score of 0.92 and a median Dice score of 0.90, with a median ASSD of 2.11 mm and a median HD95 of 4.9 mm. It should also be noted that the T1 SAG model demonstrated very similar performance in this region, with a median F2 score of 0.90, a median Dice score of 0.90, a median ASSD of 2.06 mm, and a median HD95 of 4.12 mm. Finally, the T2 SAG model was particularly effective for tumors located in the abdomen, pelvis, and trunk. For this challenging anatomical region, it achieved a median F2 score of 0.89 and a median Dice score of 0.89, which was the highest of any model in this category. It also had a median ASSD of 2.5 mm and a median HD95 of 7.5 mm.

**Table 2:** Segmentation performance of the two best-performing model configurations for each of the three main anatomical locations

Model	F2 score	Dice	Recall	Precision	ASSD	HD95
Upper extremities						
T1 SAG	0.85 (0.79–0.92)	0.88 (0.84–0.91)	0.82 (0.75–0.93)	0.96 (0.95–0.97)	1.5 (1.05- 3.4)	4.24 (3.2–11.0)
T1 COR	0.85 (0.72–0.85)	0.84 (0.71–0.88)	0.83 (0.82–0.85)	0.77 (0.69–0.95)	5.76 (3.0–26.0)	9.7 (6.7–148)
Lower extremities						
T1 AX	0.92 (0.86–0.94)	0.90 (0.85–0.94)	0.92 (0.86–0.95)	0.91 (0.82–0.96)	2.1 (0.99–6.95)	4.9 (2.6–35.3)
T1 SAG	0.90 (0.78–0.94)	0.91 (0.78–0.94)	0.91 (0.85–0.94)	0.92 (0.80–0.95)	2.1 (1.2–11.0)	4.1 (2.8–77.2)
Abd/pelvis/trunk						
T2 SAG	0.89 (0.72–0.93)	0.89 (0.74–0.94)	0.87 (0.76–0.91)	0.91 (0.74–0.97)	2.5 (1.4–8.2)	7.47 (3.3–24.2)
T2 AX	0.88 (0.85–0.92)	0.85 (0.76–0.89)	0.93 (0.91–0.95)	0.84 (0.74–0.90)	7.38 (1.7–14.8)	34.6 (5.1–98.5)

### Impact of histological subtype on model accuracy

Segmentation performance was highly dependent on the sarcoma histotype, revealing significant variability across the different subtypes (Table 3). For LMS, which was the most challenging subtype, both the T1 COR and T1 AX models achieved the best results. The T1 COR model achieved a median F2 score of 0.85 and a median ASSD of 1.56 mm, while the T1 AX model had a median F2 score of 0.88 and a median ASSD of 7.36 mm. In the segmentation of UPS, the T1 AX model was the top performer, with a median F2 score of 0.91 and a median ASSD of 2.49 mm. The T1 SAG model also performed exceptionally well, achieving a median F2 score of 0.89 and a median ASSD of 2.42 mm. For mLPS, both T1 and T2 sequences demonstrated strong performance. The T1 AX model showed high accuracy with a median F2 score of 0.92 and a median ASSD of 1.61 mm. The T2 AX model performed similarly, with a median F2 score of 0.91 and a median ASSD of 2.26 mm. For MFS, both the T1 SAG and T2 COR models performed exceptionally well, achieving the highest scores in the study. The T1 SAG model had a median F2 score of 0.93 and a median ASSD of 1.76 mm, while the T2 COR model achieved an F2 score of 0.94 and a median ASSD of 2.32 mm. Finally, for MYX, the T2 models were the most effective. The T2 SAG model showed the highest accuracy with a median F2 score of 0.91 and a median ASSD of 1.69 mm, while the T2 AX model followed closely with a median F2 score of 0.89 and a median ASSD of 5.73 mm.

**Table 3:** Segmentation performance of the two best-performing model configurations for each histologic subtype

	F2	Dice	Recall	Precision	ASSD	HD95
LMS						
T1 COR	0.85 (0.76–0.92)	0.88 (0.76–0.9)	0.83 (0.81–0.93)	0.94 (0.74–0.97)	1.56 (1.0–6.7)	3.72 (2.9–26.5)
T1 AX	0.88 (0.47–0.92)	0.86 (0.58–0.91)	0.90 (0.42–0.93)	0.89 (0.75–0.97)	7.36 (1.4–18.6)	56.59 (4.6–89.6)
UPS						
T1 AX	0.91 (0.86–0.93)	0.91 (0.85–0.92)	0.91 (0.86–0.93)	0.92 (0.88–0.96)	2.49 (1.1–4.3)	6.07 (2.4–12.7)
T1 SAG	0.89 (0.84–0.94)	0.90 (0.87–0.94)	0.91 (0.85–0.95)	0.94 (0.86–0.96)	2.42 (1.2–10.8)	50.56 (3.4–81.6)
mLPS						
T1 AX	0.92 (0.79–0.94)	0.90 (0.84–0.94)	0.93 (0.76–0.94)	0.95 (0.86–0.97)	1.61 (1.0–15)	4.12 (2.9––76.8)
T2 AX	0.91 (0.87–0.95)	0.91 (0.84–0.94)	0.94 (0.86–0.96)	0.93 (0.82–0.95)	2.26 (1.0–7.8)	4.30 (2.7–51.3)
MFS						
T1 SAG	0.93 (0.92–0.95)	0.93 (0.92–0.95)	0.94 (0.92–0.96)	0.95 (0.93–0.96)	1.76 (1.2–3.3)	3.0 (2.2–4.3)
T2 COR	0.94 (0.91–0.95)	0.92 (0.89–0.95)	0.95 (0.93–0.96)	0.91 (0.84–0.95)	2.32 (1.6–7.5)	3.93 (2.7–51.5)
MYX						
T2 SAG	0.91 (0.52–0.95)	0.84 (0.1–0.92)	0.98 (0.89–0.99)	0.84 (0.39–0.91)	1.69 (1.1–6.4)	3.0 (2.8–14.7)
T2 AX	0.89 (0.75–0.94)	0.81 (0.58–0.92)	0.97 (0.93–0.98)	0.71 (0.44–0.87)	5.73 (1.2–46.7)	11.06 (3.8–115.8)

## Discussion

The pronounced heterogeneity and broad anatomic distribution of soft tissue sarcomas pose significant challenges for diagnosis, therapy, and presurgical planning. This inherent diversity is a major obstacle for imaging research, as the scarcity of cases makes it difficult to assemble the large, subtype-specific datasets required to train robust algorithms. Although several studies have explored automated segmentation in sarcomas, most have addressed narrowly defined scenarios, such as extremity tumors, CT-based contouring for radiotherapy planning, or small single-site cohorts [15, 22, 24]. None has systematically evaluated how MRI sequence selection, imaging plane, anatomical location, and histological subtype jointly affect segmentation performance. The present study extends prior work by providing a comprehensive comparison of input strategies in a larger and more heterogeneous STS population, thereby offering practical guidance for model development and clinical implementation.

Our systematic evaluation revealed that segmentation accuracy strongly depended on tumor location and histology. For extremity sarcomas, contrast-enhanced T1-weighted images provided the most accurate delineation, consistent with their strong tumor–background contrast, whereas T2-weighted imaging was superior for tumors in the abdomen, pelvis, and trunk, where high water content offers clearer tissue differentiation when post-contrast borders are less distinct. Our top model achieved a median Dice score of 0.89, which is highly competitive when compared to state-of-the-art results for brain tumors in the BraTS challenge [17] or liver tumors in the LiTS benchmark [32], where Dice scores typically range between 0.85 and 0.92. Given the extreme heterogeneity and infiltrative nature of sarcomas compared to the more predictable morphology of organ-confined tumors, these results underscore the robustness of the nnU-Net framework in this challenging domain. Notably, our best model (T1 AX) achieved a median Dice of 0.89, outperforming recent extremity sarcoma studies [22] despite the inclusion of anatomically complex regions, underscoring the model’s robustness and potential for clinical use across diverse tumor sites.

Segmentation accuracy was also highly dependent on the tumor’s histological subtype. The models achieved their highest performance on MFS and mLPS, with F2-scores reaching as high as 0.94. This success is likely due to the distinct imaging features of these tumors; MFS is often well-circumscribed, while the high water content in mLPS creates a clear, bright signal that is easily segmented. In contrast, LMS proved to be the most challenging subtype, which is consistent with its often-infiltrative growth patterns and heterogeneous appearance that result in indistinct tumor borders. These subtype-specific results highlight a key challenge: while a generalized model can perform well, its accuracy is ultimately linked to the intrinsic imaging characteristics of each histology. A robust, universal model would therefore require a larger, more diverse dataset.

Notably, single-sequence, single-plane models outperformed multi-modal or multi-planar approaches, contradicting assumptions that more data inherently improve performance. Multi-modal (T1 + T2) fusion likely introduced “feature pollution,” where less distinct T2 boundaries degraded high-contrast T1 information. The nnU-Net’s early fusion strategy may be suboptimal for combining modalities. More advanced architectures, using attention or cross-modal fusion, could better leverage complementary data without corrupting boundary features.

Beyond technical performance, these findings offer two critical insights for the clinical translation of AI in sarcoma. First, the observation that multi-modal fusion can degrade performance due to “feature pollution” has practical implications for workflow efficiency. Rather than mandating complex, multi-sequence acquisitions that increase scan time and cost, clinical protocols could be optimized to prioritize specific high-yield sequences (e.g., T1-weighted imaging for extremities) without sacrificing segmentation accuracy. Second, the significant performance variance observed across histologies challenges the viability of a “one-size-fits-all” model for STS. Instead, our data supports the implementation of adaptive clinical workflows, where the specific segmentation algorithm is automatically selected based on the suspected or biopsy-confirmed histological subtype.

Our study has several limitations. First, as a retrospective, single-institution study, generalizability may be limited. The dataset, though large for this rare disease, remains modest and unevenly distributed across subtypes. MRI acquisition protocols varied, and not all planes were available for each case. Furthermore, our analysis was restricted to the five most common mesenchymal tumor subgroups to ensure sufficient statistical power for reliable model training. While these subtypes represent a significant portion of clinical cases, this selection excludes rarer histologies, potentially limiting the model’s immediate generalizability to the full spectrum of soft tissue sarcomas. Consequently, the current framework should be regarded as exploratory, establishing a performance baseline that must be validated and expanded to include rarer entities in future multi-institutional studies.

One limitation of this study is the absence of external validation. All data originate from a single institution, and model performance may therefore reflect site-specific imaging characteristics. Although cross-validation and a held-out test set were used to reduce overfitting, these approaches cannot fully ensure generalizability. External validation on independent multi-institutional datasets will be essential before clinical deployment. Future work should also incorporate peritumoral edema masks to refine boundary precision and avoid overestimation of tumor volume.

The limitations identified in this analysis highlight several promising directions for future research. Improving multi-modal integration will require intermediate or attention-based fusion strategies. Subtype-specific or hybrid models could enhance performance for difficult histologies like LMS and MYX. Accurate segmentation lays the groundwork for advanced radiomic and deep learning studies aimed at predicting subtype, grade, treatment response, and prognosis—transforming technical segmentation success into meaningful clinical impact.

## Conclusion

This study demonstrates that accurate, automated segmentation of soft tissue sarcomas is feasible using a deep learning framework. T1-weighted, single-plane models—particularly axial and sagittal—achieved the best overall performance, with accuracy influenced by tumor location and histology. By improving reproducibility and precision in tumor delineation, such tools can enhance surgical and radiotherapy planning, supporting personalized, function-preserving sarcoma care.

## Supplementary information

Below is the link to the electronic supplementary material.

Supplementary file1 (DOCX 17 kb)

## Data Availability

Not applicable.

## Abbreviations

MRI: Magnetic resonance imaging
T1 FS CE: T1-weighted fat-saturated contrast enhanced
T2 FS: T2-weighted fat saturated
HIPAA: Health Insurance Portability and Accountability Act
IRB: Institutional Review Board
