# Supplementary material for: Performance of deep learning-based segmentation of soft tissue sarcoma by MRI sequence, tumor type and location
Source: Skeletal Radiol. 2026 Mar 4;55(7):1539–48. doi: 10.1007/s00256-026-05178-3 (PMC13198472; doi:10.1007/s00256-026-05178-3)
Supplement: Supplementary file 1 — Supplementary file1 (DOCX 17 kb) [file 256_2026_5178_MOESM1_ESM.docx]

|  | F2 | Dice | Recall | Precision | ASSD | HD95 |
| --- | --- | --- | --- | --- | --- | --- |
| T1 AX | 0.91  (0.75 - 0.93) | 0.89  (0.8 - 0.93) | 0.91  (0.78 - 0.95) | 0.93  (0.84 - 0.97) | 2.4  (1.1 - 7.8) | 5.6  (2.9 - 56.2) |
| T1 COR | 0.89  (0.76 - 0.92) | 0.86  (0.79 - 0.91) | 0.90  (0.78 - 0.93) | 0.89  (0.74 - 0.95) | 5.7  (1.7 - 17.5) | 12.7  (3.9 - 115) |
| T1 SAG | 0.896  (0.75 - 0.94) | 0.89  (0.77 - 0.93) | 0.92  (0.83 - 0.95) | 0.92  (0.79 - 0.96) | 2.1  (1.2 - 13.3) | 4.3  (2.9 - 86) |
| T1 AX/COR/SAG | 0.9  (0.81 - 0.94) | 0.88  (0.787 - 0.925) | 0.92  (0.84 - 0.96) | 0.87  (0.74 - 0.95) | 4.5  (1.4 - 17.9) | 7.5  (3.3 - 117) |
| T2 AX | 0.88  (0.81 - 0.93) | 0.87  (0.75 - 0.92) | 0.92  (0.83 - 0.96) | 0.87  (0.72 - 0.93) | 6.1  (1.3 - 24.8) | 11.5  (4 - 102) |
| T2 COR | 0.85  (0.74 - 0.94) | 0.85  (0.72 - 0.91) | 0.88  (0.74 - 0.95) | 0.85  (0.74 - 0.933) | 5.9  (1.7 - 17.5) | 8.6  (3.7 - 107) |
| T2 SAG | 0.88  (0.74 - 0.93) | 0.88  (0.59 - 0.93) | 0.88  (0.76 - 0.96) | 0.91  (0.73 - 0.955) | 2.7  (1.6 - 15.9) | 5.1  (3.1 - 69.1) |
| T2 AX/COR/SAG | 0.87  (0.78 - 0.92) | 0.86  (0.74 - 0.91) | 0.9  (0.79 - 0.95) | 0.85  (0.71 - 0.94) | 8.4  (2.4 - 23.4) | 14.8  (5 - 141) |
| T1&T2 | 0.87  (0.74 - 0.92) | 0.86  (0.74 - 0.92) | 0.89  (0.79 - 0.94) | 0.88  (0.69 - 0.95) | 7.1  (1.8 - 24.9) | 14.2  (4.2 - 136) |

**Table S1**: Aggregated segmentation performance for all model configurations across all histological subtypes and tumor locations.
